# Age-related changes in murine bladder structure and sensory innervation: a multiphoton microscopy quantitative analysis

**DOI:** 10.1007/s11357-016-9878-1

**Published:** 2016-01-29

**Authors:** Anna Schueth, Bart Spronck, Marc A. M. J. van Zandvoort, Gommert A. van Koeveringe

**Affiliations:** 1 Department of Urology, Maastricht University Medical Center, P.O. Box 5800, 6202 AZ Maastricht, the Netherlands; 2 School for Mental Health and Neuroscience (MHeNS), Maastricht University, P.O. Box 616, 6200 MD Maastricht, the Netherlands; 3 Department of Biomedical Engineering School for Cardiovascular Diseases (CARIM), Maastricht University, 6229 ER Maastricht, the Netherlands; 4 Department of Genetics and Cell Biology – Molecular Cell Biology, School for Cardiovascular Diseases (CARIM), Maastricht University, 6229 ER Maastricht, the Netherlands; 5 Institute for Molecular Cardiovascular Research (IMCAR), RWTH University of Aachen, Pauwelsstrasse 30, 52074 Aachen, Germany

**Keywords:** Urinary bladder, Sensory innervation, Mice, Multiphoton microscopy, Aging

## Abstract

Our study aimed to examine and quantify age-related structural alterations in the healthy mouse bladder using ex vivo two-photon laser scanning microscopy (TPLSM). Freshly dissected bladders from 25-, 52-, and 85-week-old C57bl/6J mice were examined, and morphological analyses and quantification of cell layers and nerves were performed. The numbers of stretched, curled, branched, and total number of nerves in volume units of the stained muscle layer were quantified. We observed differences in the bladder wall architecture and innervation with age. Especially in 85-week-old mice, age-related changes were found, including detachment of urothelial cells and an increase in connective tissue, intermingled with the smooth muscle fibers in the muscle layer (collagen-smooth muscle ratio of 1.15 ± 0.29). In 25- and 52-week-old mice, the collagen-smooth muscle ratios were 0.20 ± 0.04 and 0.31 ± 0.11, respectively, and a clear separation of collagen and muscle was observed. The overall number of nerves and the number of curled nerves were significantly higher in the 85-week-old mice (74.0 ± 13.0 and 25.9 ± 4.8, respectively), when comparing to 25-week-old mice (26.0 ± 2.7 and 6.7 ± 1.2, respectively) and 52-week-old mice (43.8 ± 4.3 and 22.1 ± 3.3, respectively). Significant age-related alterations in bladder morphology and innervation were found, when comparing freshly dissected bladder tissue from 25-, 52-, and 85-week-old mice. The higher number of curled nerves might be an indication of an increased neurotransmitter release, resulting in a higher nerve activity, with a part of the nerves being possibly mechanically impaired. This study shows that two-photon laser scanning microscopy of healthy aging male mice is a useful method to investigate and quantify the age-related changes in the bladder wall.

## Introduction

Within the elderly population, malfunction and age-related changes of the lower urinary tract (LUT) are very common and accompanied by a decrease in bladder capacity, as well as an increasing detrusor overactivity (Elbadawi et al. 1998; Elbadawi et al. 1993; Lluel et al. 2000). Overactive bladder syndrome (OAB) is an example of a LUT disorder that is characterized by impaired bladder storage. OAB is thought to be multifactorial (Natalin et al. 2013; Daly et al. 2014) and characterized by a combination of symptoms, including frequent micturition, urgency, and sometimes urinary incontinence (UI) and nocturia (Daly et al. 2014; Yoshida 2009).

In aged men, OAB symptoms are often associated with a LUT obstruction based on benign prostatic hyperplasia, while in the majority of women and a smaller part of men, its cause is idiopathic in nature (Gomez and Andrianne 2014). Resultant UI within the elderly population is responsible increasing health care costs for care and treatment in the coming decades (Natalin et al. 2013; Yoshida 2009).

Normal functions of the healthy urinary bladder are storage and expulsion of urine. During micturition, with a complex pattern of efferent and afferent signaling, the detrusor muscle is contracting and the urethral outlet relaxing (Lluel et al. 2000). The autonomic (parasympathetic and sympathetic) and somatic nervous system innervate the LUT. The parasympathetic nervous system mediates contraction of the detrusor muscle (micturition), while the sympathetic nervous system contributes to urine storage (relaxation of the detrusor muscle and contraction of the bladder neck and urethra) (Fowler et al. 2008). Two types of afferent nerve fibers can be found in the LUT: (1) small myelinated Aδ fibers, which respond to changes in tension in the bladder wall, and (2) unmyelinated C fibers, which respond to chemical or cold irritation (Kenton et al. 2007).

Several factors are leading to geriatric voiding dysfunction, such as (1) bladder wall fibrosis, (2) a decline of detrusor contractility and urethral tone, and (3) a decline in the sensation of having to urinate until the need is suddenly very urgent (Elbadawi et al. 1993; Siroky 2004; Pfisterer et al. 2006; Yoshida 2009). MRI studies in women by Griffiths et al. (2007) showed that with increasing age, brain regions involved in bladder control, including right insula, show decreased responses.

Studies into age-related morphological changes in the bladder wall are scarce. An electron microscopy study of the urinary bladder from senile female rats showed mucosal damage, increased collagen deposition, and degeneration of the smooth muscle fibers of the detrusor muscle (Al-Motabagani 2005). In another study, alterations of urothelial sensory signaling, with bladder hypersensitivity and hyperactivity during aging, were reported (Daly et al. 2014).

The aim of our study was to investigate and quantify age-associated structural changes in morphology of the bladder wall and the innervation therein.

## Materials and methods

### Animals

Experimental protocols were approved by the animal ethics committee of Maastricht University and were carried out according to the institutional guidelines and reported in accordance with the ARRIVE guidelines. C57bl6J male mice (25-, 52-, and 85-week-old mice, each *n* = 5) were housed individually within a temperature-controlled environment with 12-h light/dark cycle, standard chow and water available ad libitum.

### Tissue preparation

**Fig. 1:**
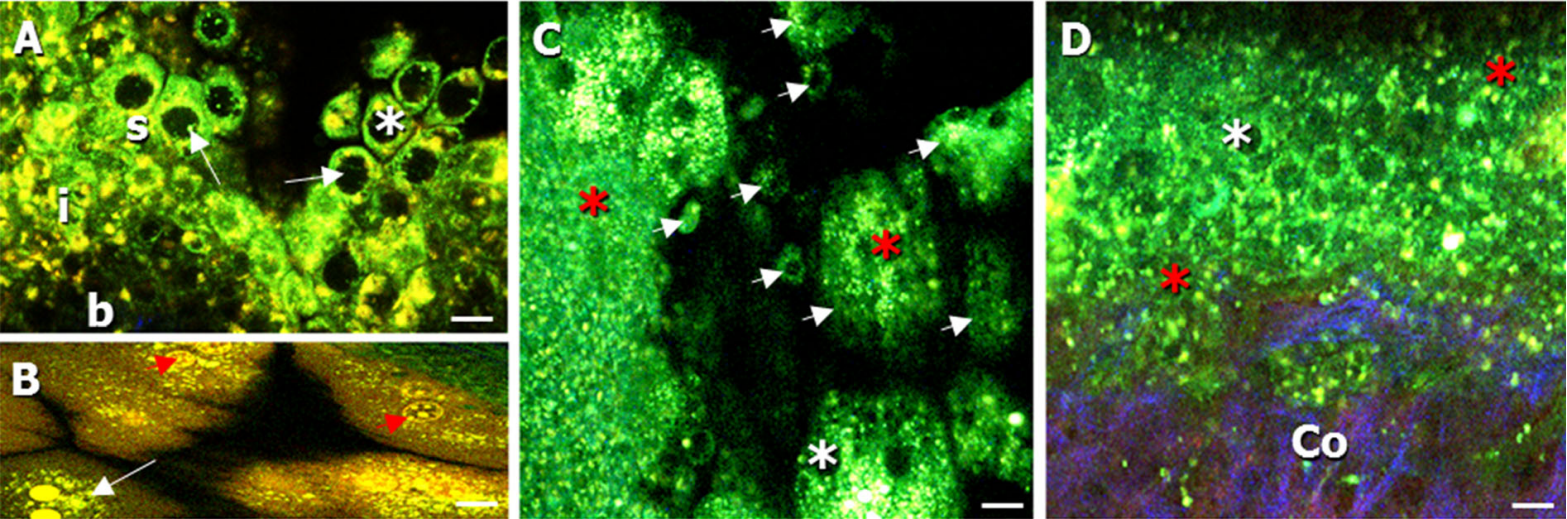
Autofluorescent, label-free images of the bladder mucosa (imaged from the urothelial side) of **a**, **b** 25-week-old, **c** 52-week-old, and **d** 85-week-old mice. **a** Transepithelial lining of the urothelium (*s* superficial layer, *i* intermediate layer, and *b* basal layer). Cytoplasm of urothelial cells (*white arrow*) was visible in the green channel, with non-fluorescent nuclei (*white asterisk*). **b** Typical hexagonally-shaped umbrella cells of the superficial urothelial layer (*white arrow*), showing cell components (*red arrowhead*). **c** Detachment of superficial urothelial cells from the epithelial lining (*white arrow heads*) and degeneration of urothelial cells (*red asterisk*). **d** Increased degeneration of urothelial cells (*red asterisk*). Cells appeared more deformed and showed a loss of cytoplasm. The multi-layered epithelial lining of the urothelium was distorted; the underlying collagen in the lamina propria/sub-urothelium was visible (*Co*). Collagen was visualized in the *blue* channel due to second harmonic generation. *Scale bars*, 20 μm

**Fig. 2:**
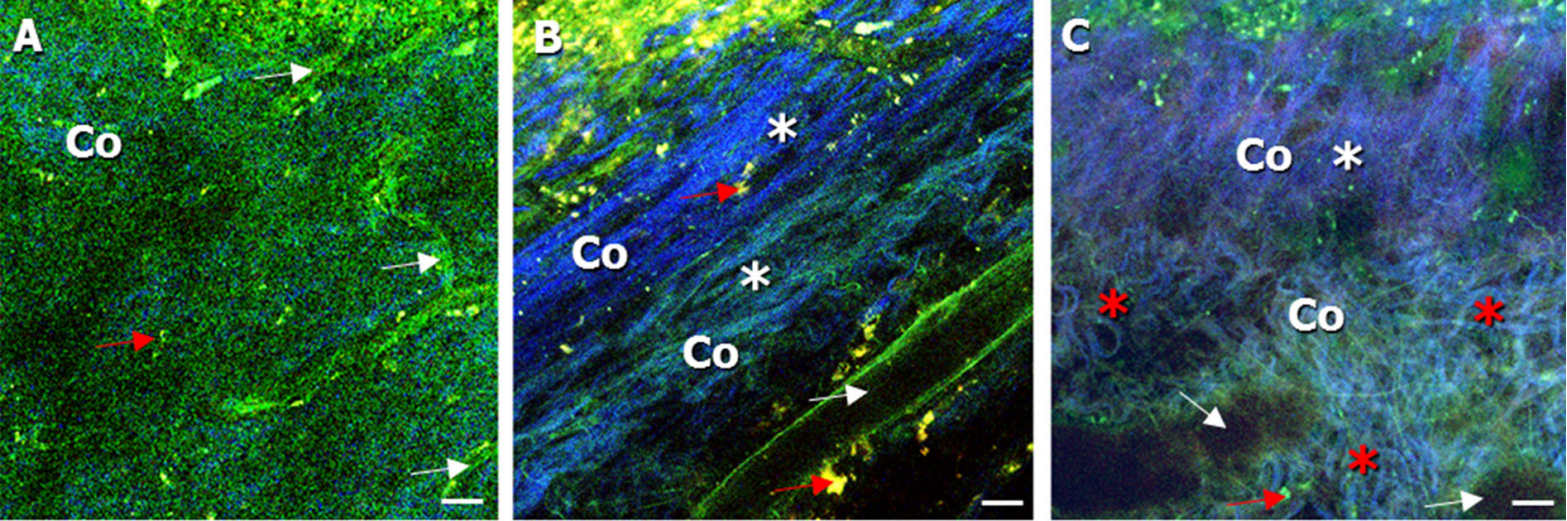
Autofluorescent, label-free images of the lamina propria (*LP*) (imaged from the urothelial side) in the bladder wall of **a** 25-week-old, **b** 52-week-old, and **c** 85-week-old mice. **a** Collagen, below the urothelium, could be seen in the second, spectral channel (*Co*). Vessel structures with a diameter ranging from 10 to 40 μm were found (*white asterisk*). **b** Collagen could be detected with a strong second harmonic generation signal (*Co*, *white asterisk*). Also, blood vessel could be seen in the LP (*white arrow*). **c** An increase of amount and density of collagen was noticeable, reaching from the sub-urothelium, throughout the LP to the smooth muscle layer (*Co*). The connective tissue appeared multi-layered, with a layer existing of rather straight-shaped collagen (*Co*, *white asterisk*) bundles (20–40 μm), followed by a thick layer of curled collagen (*Co*, *red asterisk*) bundles (40–80 μm). Vessel structures in the LP were more dilated and were showing a diameter of up to approx. 70 μm (*white arrow*). Macrophages were also found (*red arrow*) and confirmed with MOMA-1 immuno-histochemical staining. *Scale bars*, 20 μm

**Fig. 3:**
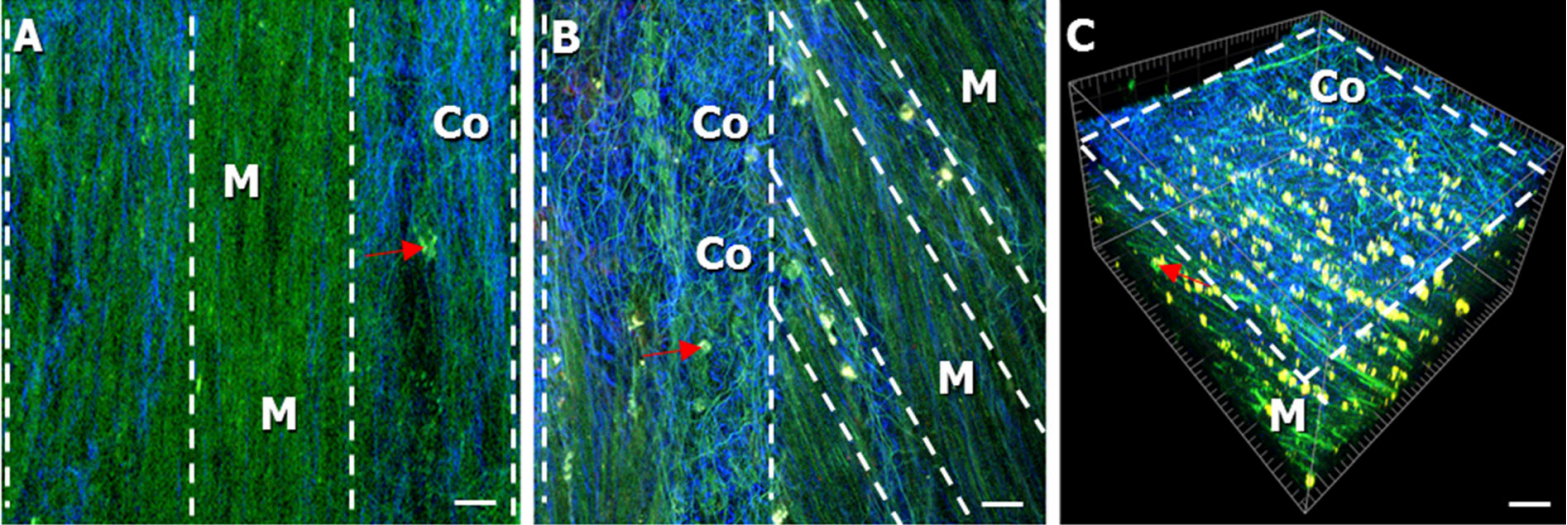
Label-free images (imaged from the adventitial side) of the muscle layer and collagen-smooth muscle ratio in the bladder wall of **a** 25-week-old and **b**, **c** 85-week-old mice. Collagen was seen in the *blue* spectral channel (indicated by *Co* and *white broken lines*) and could be separated from the muscle layer, seen in the *green* spectral channel (indicated by *M*). **a**–**c** The amount of *blue* connective tissue (*Co*) increased significantly (*p* = 0.001) in 85-week-old mice in comparison to that in 25-week-old mice. It appeared more intermingled with the *green* bundles of the muscle layer (*M*). **a** is taken from a z-stack of the muscle layer. LP and urothelium can be found below the muscle layer in the z-stack, but not shown in the image. **c** 3D visualization of **b**: tissue volume showing that the collagen-smooth muscle ratio of the aged mice (collagen indicated by *broken lines* and *Co*, muscle indicated by *M*), which also increased significant (*p* < 0.001) in contrast to the 25-week-old mice. Macrophages were found and indicated by a *red arrow*. *Scale bars*, 20 μm

The preparation of fresh urinary bladders for two-photon laser scanning microscopy (TPLSM) imaging has been described in a previous publication (Schueth et al. 2014). Prepared, opened bladders were placed in a petri dish and submerged under saline solution for TPLSM imaging. Due to the small size of the mouse bladder, only the dome was imaged. Indeed, the lateral wall, which has been described in most studies, was not suitable for this study since they were not located in the central part of the preparation and thus not accessible for the imaging equipment. Images were taken while imaging both from the urothelial (Figs. 1 and 2) and adventitial side (Figs. 3 and 5).

### Staining

For TPLSM imaging, samples were prepared either label-free, visualizing AF controls, or with a dye, visualizing cell nuclei with SYTO 13 (Life Technologies Europe BV, Bleiswijk, NL) and elastin with sulforhodamine B (Life Technologies Europe BV). Sulforhodamine B was diluted in HBSS (Life Technologies Europe BV) without phenol red, to a final concentration of 5 μM; SYTO 13 was diluted in PBS (B.Braun, Melsungen, Germany) to a final concentration of 2.5 μM. After removing excess saline solution to avoid further dilution of the marker, sulforhodamine B and SYTO 13, respectively, were added to the living, fresh bladder sample and each of the agents was incubated for 30 min at 4 °C. During imaging, tissue was submerged under physiological saline solution.

### Imaging

For imaging experiments, a two-photon laser scanning microscope (Leica TCS SP5 MP, Leica Mikrosysteme Vertrieb GmbH, Wetzlar, Germany), equipped with a HCX APO L 20×/1.00 W water immersion objective was used. Working distance of the objective was 2 mm, and the excitation source was a 140 fs-pulsed Ti:sapphire laser (Chameleon Ultra II, Coherent Inc., Santa Clara, CA, USA), mode-locked at 800 nm. To avoid photobleaching and tissue damage, laser power was kept as low as possible (max. 180 mW at the sample surface). Fluorescence emission was detected using photomultiplier tubes (Hamamatsu, R9624, Japan) in three wavelength ranges: 385–489 nm (blue), 489–563 nm (green), and 568–700 nm (red). Image acquisition settings (resolution, scan speed, etc.) are elaborated in Table 1. Imaging settings were optimized for detecting both autofluorescent (AF) structures and stained structures.

**Table 1:** TPLSM image acquisition settings

Mode	Scan field resolution	Scan field size [μm × μm]	Pixel size [μm]	Line rate [Hz]	Line averaging	Frame rate [Hz]
Examination	512 × 512	738 × 738	1.4	400	1	0.8
Single slice recording	1024 × 1024 or 512 × 512	434 × 434 or 738 × 738	0.4 or 1.4	50 or 100	2	0.05–0.1
Stack recording	1024 × 1024 or 512 × 512	434 × 434 or 738 × 738	0.4 or 1.4	100 or 200	2	0.0–0.2

Stacks were recorded with a slice spacing of 1 μm

### Image analysis

Images were recorded and analyzed with Leica Application Suite Advanced Fluorescence (Leica Microsystems). Further image processing was performed using ImageJ (W.S. Rasband, National Institutes of Health, Bethesda, MD, USA).

### Image processing

#### 3D reconstructions

3D reconstructions were created using ImageJ (Brightest Point method, no opacity, total rotation of 360°).

#### Determination of collagen and muscle layer thickness and collagen-smooth muscle ratio

Determination of the layer thickness, collagen, and muscle layer respectively was done with viewing z-stacks (stained or unstained tissues), ideally imaged from the adventitial side, with Leica LAS AF. Scrolling through the z-stacks, with a slice spacing of 1 μm, allowed determination of the thickness of the tissue layer (in μm) from the sidebar of the software. Subsequently, the collagen-smooth muscle ratio (for each z-stack) was calculated.

#### Quantification of nerves in the muscle layer (in defined volumes)

For nerve quantification, a defined sub volume of approx. 8 × 10^5^ μm^3^ was selected in every z-stack of the stained muscle layer. Stretched, branched, curled, and the total number of nerves within the volume were counted. Nerves were characterized as “curled” if they showed a radius of curvature <10 μm, as “stretched” if they showed no significant curvature, and as “branched” if they were branching from another (typically “stretched”) nerve. The “total” number of nerves (either stretched or curled, branching points were calculated separately) was counted manually by viewing each image of the defined z-stack volume and “following” the nerve in depth. An overview of nerve types in the muscle layer, with their typical shapes, can be found in Fig 5.

Images were recorded, while imaging from the adventitial side using Sulforhodamine B stained tissue, in order to discriminate between stained elastic fibers (red spectral channel) and nerve fibers (green spectral channel).

#### Statistics

Non-parametric one-way analysis of variance (ANOVA) amongst age groups was performed using Kruskal-Wallis tests. Individual age groups were compared using Wilcoxon rank-sum tests. Age trends over groups were quantified by nonparametric (Spearman) correlations. *P* values <0.05 were considered statistically significant. Unless indicated otherwise, numbers are presented as mean ± standard error of the mean (SE).

## Results

### Age-related changes of cell and layer morphology of the bladder wall

**Fig. 4:**
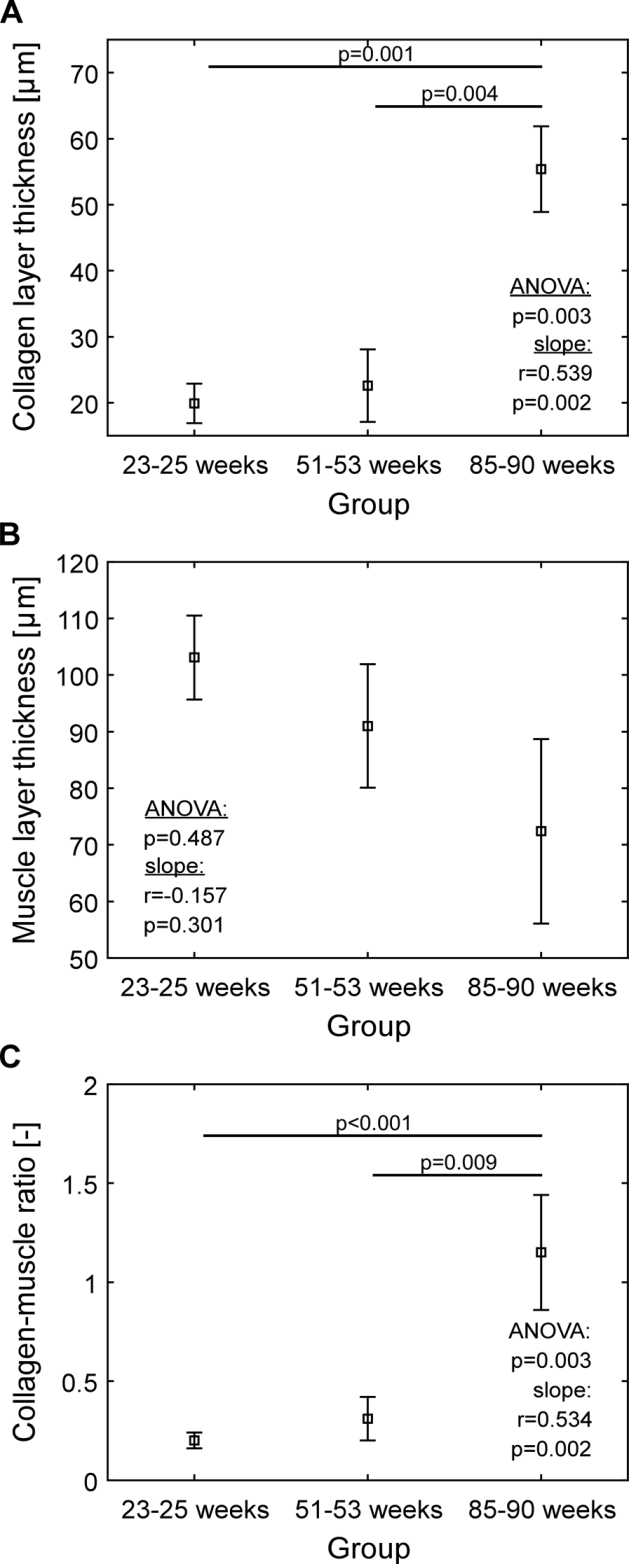
Quantification of layer thickness: **a** Collagen layer, **b** muscle layer, **c** collagen-smooth muscle ratio. *Squares* indicate means, *whiskers* indicate ±1SE. *SE* standard error of the mean. In producing panel **c**, firstly, collagen-muscle ratios of all stacks were computed. Secondly, from thus array of ratios, mean and the ±SE were computed

**Fig. 5:**
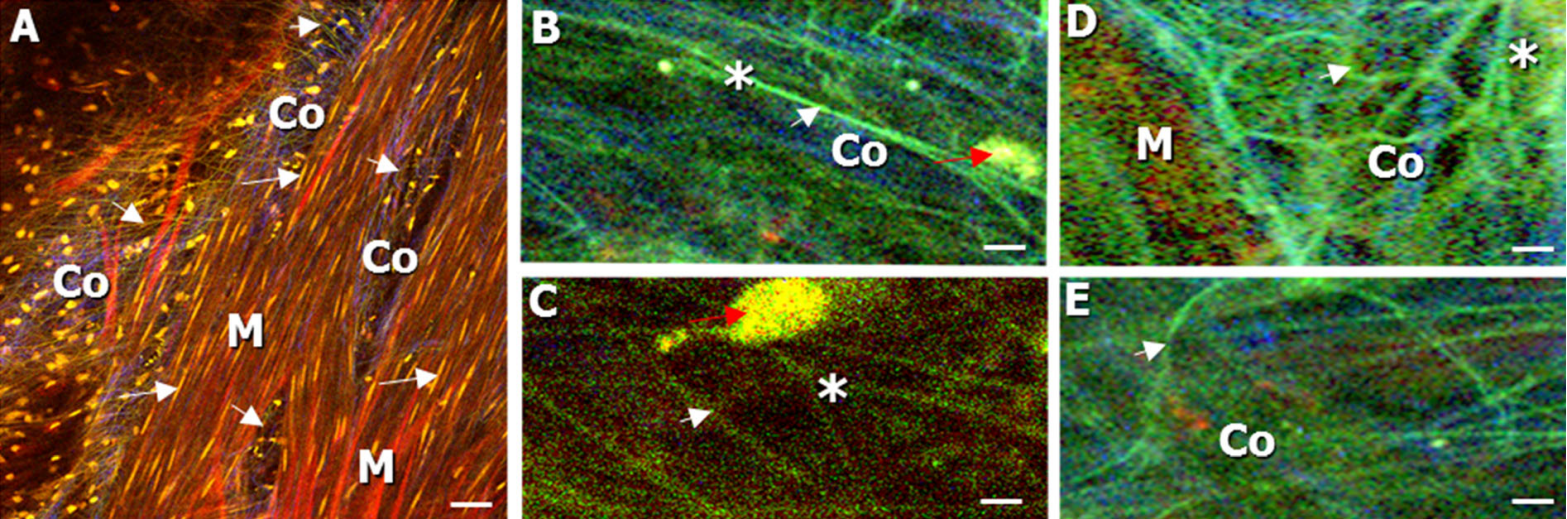
Images of label-free nerve fibers (imaged from the adventitial side) in the stained muscle layer of the bladder wall. Nerves (*white arrowhead*) and cell nuclei (SYTO 13 staining) of macrophages (*red arrow*) and smooth muscle cells (*white arrow*) could be detected in the *green* channel. Collagen (*Co*) was seen in the *blue* channel, and stained muscle fibers (*M*, Sulforhodamine B staining) in the *red* channel. **a** Straight-shaped muscle bundles (*M*) with smooth muscle cells (*white arrow*) and nerves (*white arrowhead*). **b**, **c** Magnification of stretched nerves (*white arrowhead*), showing a branching point (*asterisk*). **d**, **e** Magnification of curled nerves (*white arrowhead*) with branching point (*asterisk*). *Scale bars*, 20 μm

**Fig. 6:**
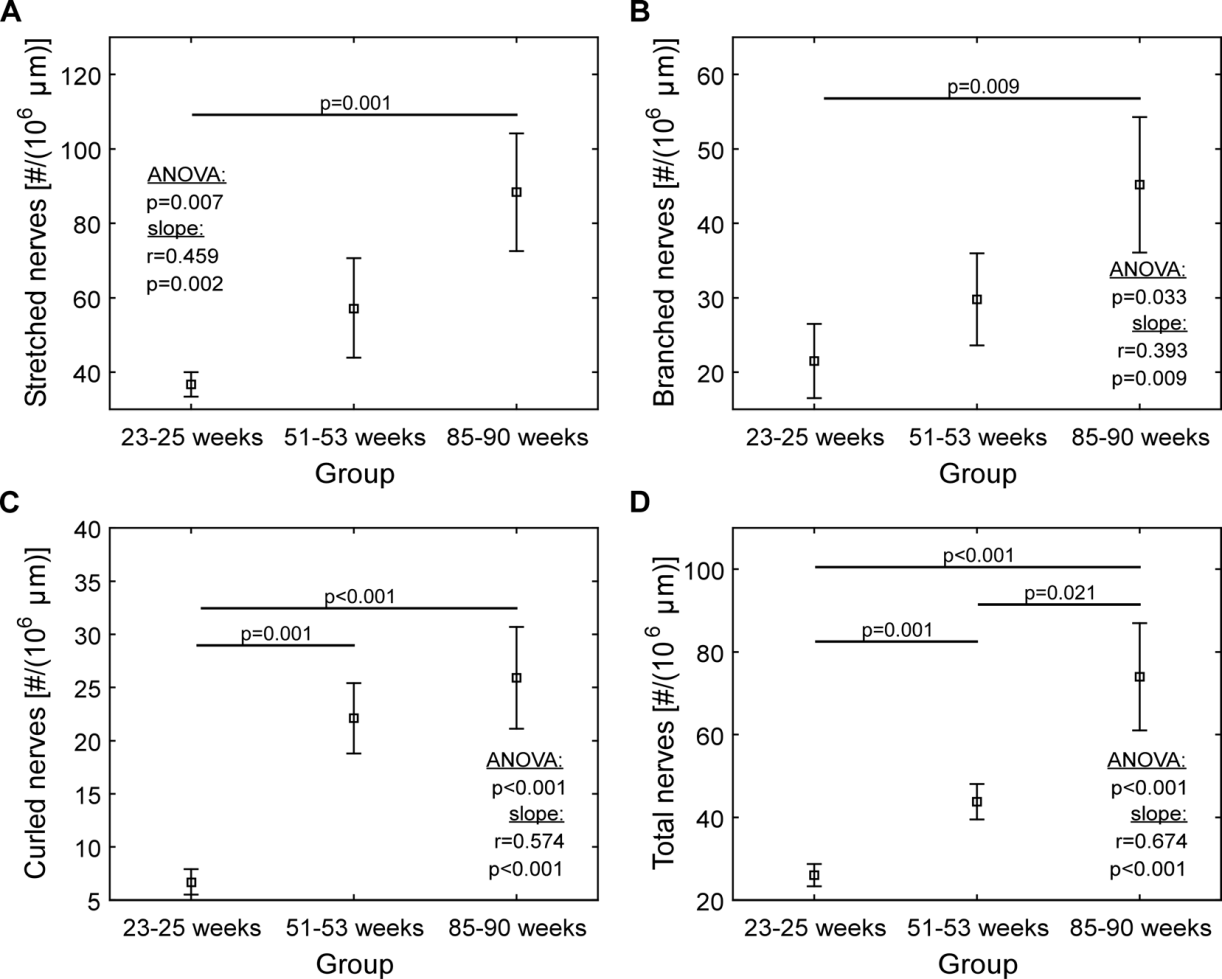
Quantification of nerves in the muscle layer of the dome region: **a** stretched nerves, **b** branched nerves, **c** curled nerves, and **d** total number of nerves. *Squares* indicate means, *whiskers* indicate ±1SE. *SE* standard error of the mean

Four layers, i.e., urothelium (Fig. 1), lamina propria (LP, Fig. 2), muscle layer (Figs. 3, 4, 5 and 6), and adventitia/serosa (Figs. 3 and 4), were clearly visible in the samples from all groups.

### Urothelium

The urothelium is a multi-layered, transitional epithelium, built up from a superficial (Fig. 1a(s)), an intermediate (Fig. 1a(i)), and a basal layer (Fig. 1a(b)). In 25-week-old mice, a transepithelial lining of the urothelium was identified (Fig. 1a).

The urothelium was visible in the green spectral channel due to AF. The hexagonally shaped umbrella cells (Fig. 1a, b (arrow)) of the superficial urothelial layer had a diameter of approximately 15–20 μm (Fig. 1a, b), with non-fluorescent nuclei (Fig. 1a–d (white asterisk)) surrounded by strong, green fluorescent cytoplasm. In the urothelium of 52-week-old mice, detachment of superficial cells (Fig. 1c (arrowheads)) from the superficial layer was noticeable in combination with a degeneration of the cells (Fig. 1c (red asterisk)). Urothelial cells in the 85-week-old mice showed an even higher degree of degeneration, and the cells, especially of the superficial layer, appeared more deformed and showed a loss of cytoplasm (Fig. 1d (red asterisk)). The typical and folded epithelial lining of the urothelium was distorted, and the underlying collagen in the LP was visible (Fig. 1d (Co)).

### Lamina propria (LP)

In 25-week-old mice, we could detect a collagen layer with a thickness of approx. 20–30 μm in the lamina propria, situated below the urothelium and seen in the blue channel (Fig. 2a (Co)). In the LP of 25- and 52-week-old mice, blood vessels with diameters from 10 to 40 μm were observed (Fig. 2a, b (white arrow)). When examining bladder tissue from 85-week-old mice, the LP was already visible through the distorted urothelial layer in the bladder mucosa (Fig. 2c (Co)). Additionally, blood vessels in the LP were more dilated and showed a diameter of up to approx. 70 μm (Fig. 2c (white arrow)). Moreover, we could see an increase of the amount and density of collagen, reaching from the sub-urothelium throughout the LP to the smooth muscle layer (Fig. 2c (Co, white asterisk)). The connective tissue appeared multi-layered, with a layer consisting of rather straight-shaped collagen bundles (at depth of 20–40 μm) (Fig. 2c, (Co, white asterisk)), followed by a thick layer of curled collagen bundles (at depth of 40–80 μm) (Fig. 2c (Co, red asterisk)).

Macrophages were also found in the connective tissue of the LP, in all three groups of mice (Fig. 2 (red arrow)). In our previous study, macrophages were confirmed with MOMA-1 immuno-histochemical staining (Schueth et al. 2014).

### Thickness of collagen and muscle layers

In 25- and 52-week-old mice, we could see the muscle layer (103.1 ± 7.4 μm and 91.0 ± 10.9 μm, respectively, Fig. 4b) being organized in a network of smooth muscle fibers (regular longitudinal and circular directions) (Figs. 3a and 5a, indicated by M). The thickness of the collagen layer was 19.9 ± 3.0 μm and 22.6 ± 5.5 μm, respectively (Fig. 4c) and the collagen-smooth muscle ratio 0.20 ± 0.04 and 0.31 ± 0.11, respectively (Fig. 4a).

In the 85-week-old mice, collagen and smooth muscle layers were rather intermingled (Fig. 3b, c indicated by M and Co), and the smooth muscle fibers were less organized as compared to that in the younger mice and fibers running mainly in a longitudinal way (Fig. 3a (M)). In the aged mice, we saw a collagen-smooth muscle ratio of 1.15 ± 0.29 (Fig. 4a), a thickness of the collagen layer of 55.4 ± 6.5 μm and of the smooth muscle layer of 72.4 ± 16.3 μm. This collagen-smooth muscle ratio is significantly increased compared to that in both 25- (*p* < 0.001) and 52-week-old mice (*p* = 0.009). Also, the collagen layer in 85-week-old mice (was significantly thicker than both in 52-week-old mice (*p* = 0.004) and 25-week-old mice (*p* = 0.001; Fig. 4c).

Muscle layer thickness did not decrease significantly with age (ANOVA *p* = 0.487, correlation *p* = 0.301). Collagen layer thickness, however, did increase with age (ANOVA *p* = 0.003, correlation *p* = 0.002), as did the collagen-smooth muscle ratio (ANOVA *p* = 0.003, correlation *p* = 0.002).

### Quantification of nerve fibers in the dome region

Imaging the bladder stained with sulforhodamine B and SYTO 13, we could identify the muscle layer (Figs. 3 and 5, indicated by M), the containing elastic fibers, and nerves (Fig. 5a (white arrowhead)). The nerves (Fig. 5 (white arrowhead)) could clearly be discriminated from sulforhodamine B-stained muscle fibers (marker for elastic fibers, see “Staining” section), as the label-free nerves appeared in the green and the stained elastic fibers in the red spectral channel. Defined volumes of the muscle layer of the bladder wall from all three groups of mice were analyzed (see “Quantification of nerves in the muscle layer (in defined volumes*)*” section). Stretched, curled, and branched nerves in different quantities were found (Fig. 5 (white arrowhead and asterisk); Fig. 6).

The numbers of nerves (stretched, branched, curled and total) in the 85-week-old mice were all significantly different from these numbers in 25-week-old mice (Fig. 6). Specifically, the number of curled nerves showed the largest difference (Fig. 6c, 25.9 ± 4.8 and 6.7 ± 1.2, respectively). The total number of nerves, seen in the aged mice, was also significantly higher than the total number of nerves in 52-week-old mice (Fig. 6d, 74.0 ± 13.0 and 43.8 ± 4.3). When comparing 25- and 52-week-old mice, both the number of curled (Fig. 6c, 06.7 ± 1.2 and 22.1 ± 3.3, respectively) and total number of nerves (Fig. 6d, 26.0 ± 2.7 and 43.8 ± 4.3, respectively) were significantly different.

## Discussion

Although it has been stated that there is a correlation between old age and LUT disorders, like OAB and UI, aging of the urogenital tract remains poorly understood (Gibson and Wagg 2014; Haferkamp and Elbadawi 2004). Possible main factors leading to geriatric voiding dysfunction are bladder wall fibrosis, decreased detrusor contractility and urethral tone, as well as an abnormal bladder sensation (urgency) (Elbadawi et al. 1993; Siroky 2004; Pfisterer et al. 2006; Yoshida 2009). Also, the elderly population experiences a decrease of deep sensation of vibration and proprioception, as well as superficial sensation (touch, temperature, pain) (Iwamoto et al. 2013). In the bladder wall of the elderly, an abnormality in nervous supply/innervation has been found before (Yoshida 2009).

In our study, we showed age-related changes in bladder morphology and innervation, when comparing freshly dissected bladder tissue from 25-, 52-, and 85-week-old mice.

The structural damage to the urothelium (detachment, degeneration, and loss of cytoplasm) found in older mice could lead to a loss of its barrier function and an increased permeability for water, urea, and harmful matter. This would result in a higher chance of cystitis and inflammation, with consequent urgency, frequency, and pain (Lavelle et al. 1997; Parwani et al. 2004; Jezernik et al. 2000; Keay et al. 2014; Al-Motabagani 2005).

Also, in the 85-week-old mice, we saw that collagen and muscle tissue were intermingled (Fig. 3b, c, indicated by M and Co), while they were clearly separated in two layers in 25- and 52-week-old mice. Most probably, an aged-induced efferent and afferent denervation of the bladder is responsible for the increase in fibrosis around and inside the detrusor muscle (Elbadawi et al. 1998). Collagen has the function of support and protection of the bladder, avoiding over-distension under high-pressure conditions (Murakumo et al. 1995). The general denervation and a denervation-induced hypertrophy may be causing an increased nerve growth factor (NGF) expression and an upregulation of muscarinic receptors (Braverman et al. 1998), which in turn can be responsible for detrusor overactivity (Ochodnicky et al. 2011; Michel 2015).

Mostly aging studies of the urinary bladder are done in rats. Lluel et al. (2000) found no fibrosis in female rats, in contrast with the fibrosis common in the human elderly population. In line with the latter, we saw a significant increase of connective tissue with age, accompanied by a significant increase of the collagen-smooth muscle ratio (trend, *p* = 0.002). Also, we could detect a decrease in smooth muscle cell layer thickness, which was not significant.

These findings are in line with those from Al-Motabagani (2005), who described an increase of connective tissue with a separation of irregularly arranged muscle bundles, in aged female rats. The excess of collagen in the detrusor muscle is characteristic for bladder fibrosis, which is common amongst the elderly (Holm et al. 1995), and now also detected in the aged mice (Fig. 4a). Detrusor fibrosis could be the cause of geriatric voiding dysfunction with impairment of the detrusor contractility and even of bladder failure. Siroky (2004) described an interrelationship between reduced blood flow in the bladder with resulting hypoxia and ischemia, leading to bladder wall fibrosis as well as low bladder compliance and reduced contractility. Through mechanisms described above, increased NGF expression and muscarinic receptor upregulation, in the process of ischemia and denervation also detrusor overactivity, often occurs in the course of the aging process (Sagawa et al. 2013). But the other way around, high bladder pressures due to a fibrotic and therefore less compliant bladder wall, can also lead to bladder ischemia. Although the changes in murine muscle layer thickness are not significant, a decreasing trend with age is visible. This could, together with the increasing collagen thickness, the increased collagen-smooth muscle ratio, and the increased mixture of collagen and smooth muscle, relate to earlier functional findings of an increased detrusor underactivity (DU) with age (Sagawa et al. 2013). A reduced contractile ability of the detrusor leads to an incomplete emptying of the bladder (van Koeveringe et al. 2014). The etiology of DU is thought to be multi-factorial, including aging, bladder outlet obstruction, and neurological diseases (Drake et al. 2014; van Koeveringe et al. 2014). Although there has been an increased interest in DU, no effective treatment has been found yet (Chapple et al. 2015). More (morphological) research on bladder level may shed new light on the development of DU with aging and a possible association of DU and aging.

To date, there have been only few studies investigating the effect of aging on the interplay between bladder function, neural (peripheral, central, motor and sensory), activity and communication. Mills et al. (2000) compared the physiological properties and innervation pattern of human detrusor muscle strips, using bladder biopsies obtained from patients with idiopathic detrusor instability (IDI) and post-mortem controls (median age range approximately 50 years). The analysis of 10-μm thin histological sections with classical fluorescence microscopy revealed a reduced number (denervation) and an altered distribution of acetylcholinesterase-positive nerves in the detrusor of IDI patients (Mills et al. 2000). Although, in currently available literature, a denervation of the (pathological) detrusor is reported more regularly, in the here presented study, we found an overall increase in the number of nerves in the aged mice (Fig. 6d). This finding is also in contrast with earlier animal research where a decrease of nerve supply, e.g., denervation, was shown in aged subjects (Sagawa et al. 2013; Gilpin et al. 1986). In these above mentioned studies, only specific nerves were stained, while in our study, nerves were detected and quantified by autofluorescence. We cannot confirm that all the nerve-like structures found with autofuorescence are in fact functional. It might very well be that we also identified damaged, non-functional nerves, while in the studies mentioned above, only functional nerves have been stained. Moreover, functional experiments by Daly et al. (2014) have revealed an increase in afferent activity related to aging, which corroborates the morphological findings in our study. Thus, in order to distinguish between functional, non-functional, and different types of nerves additional differentiating, stainings related to function are needed.

When looking at nerve morphology, the number of curled nerves in the muscle layer was four times higher (*p* < 0.001, Fig. 6c) in the 85-week-old mice, when comparing with 25-week-old mice. The higher numbers of curled nerves in the aged mice might suggest a different nerve activity. Previous work in the intestine has shown a relationship between morphological characteristics of nerve terminals and their function and activity (Berthoud et al. 2004). Another explanation might be that the nerves are mechanically impaired, e.g., stretched and then released, because of multiple stretch and release episodes.

In addition, the authors speculate that the findings of both a damaged urothelium in older mice, as well as an increase of the number of overall nerves in the muscle layer, indicate a malfunctioning communication of the urothelium with the underlying “sensory web.” This leads to malfunctioning coordination of contractions of the detrusor muscle and the relaxation of urethra for urinary storage and micturition. Another possible effect is a not fully functioning detrusor muscle, resulting in involuntarily contractions, as is very common amongst the elderly to experience urgency, often combined with UI and nocturia (Yoshida 2009).

Gosling et al. (1986) showed a significant reduction of autonomic nerves in the human detrusor muscle of obstructed patients, in comparison with a non-obstructed control group. In the human situation, it has been shown that aging in combination with infravesical obstruction is more likely to result in fibrotic changes of the bladder (Gosling 1997). In male mice, however, an anatomical obstruction by the prostate in a natural course of aging is unlikely due to the anatomical location of the prostate in mice. Therefore, the most likely cause of the changes in our study is aging as such.

In this study, we have focused on morphological changes, associated with old age and we quantified the number of nerves in the muscle layer of the murine bladder wall. We found cell detachment within the urothelial layer and an increase in collagen, collagen-smooth muscle ratio, the overall number of nerves, and number of curled nerves in the aged mice. Further analysis of the complete bladder wall would provide better insight in age-related alterations of bladder motor-innervation and motor-sensory signaling.

Moreover, the present study showed that TPLSM of healthy male mice bladders is a useful method to investigate bladder aging, being a basis for follow-up studies, like intravital imaging, to investigate age-related differences in bladder function.
